# Aging Processes and Their Influence on the Mechanical Properties of Printable Occlusal Splint Materials

**DOI:** 10.3390/polym15234574

**Published:** 2023-11-29

**Authors:** Jan Raffael Rosello Jimenez, Florian Fuchs, Leonie Schmohl, Michaela Schulz-Siegmund, Andreas Koenig

**Affiliations:** 1 Department of Prosthodontics and Material Sciences, Leipzig University, 04103 Leipzig, Germany; 2 Private Practice, Zahnarztpraxis Jan Rosello, 04654 Frohburg, Germany; 3 Institute of Pharmacy, Pharmaceutical Technology, Leipzig University, 04317 Leipzig, Germany

**Keywords:** 3D printed, tensile strength, elastic modulus, Young’s modulus, Vickers hardness, thermocycling, bite splints

## Abstract

Three-dimensional (3D)-printed occlusal splints are becoming more prevalent in the treatment of tooth substance loss due to their fast and cost-effective production. The purpose of this in vitro study was to investigate whether the mechanical properties (tensile strength—TS, modulus of elasticity in tension—ME, and Vickers hardness—HV) vary between the materials (printed dimethacrylate-based resins: Keyprint KeySplint soft—KEY, Luxaprint Ortho Plus—LUX, V-Print splint—VPR, printed methacrylate-based resins Freeprint splint 2.0—FRE, and milled methacrylate-based material, CLEAR splint—CLE), and the influence of aging processes (extraoral storage conditions and nightly or daily use) was examined. The printed methacrylate-based resins (FRE, LUX, and VPR) had much higher TS (43.7–48.5 MPa compared to 12.3–13.3 MPa), higher ME (2.01–2.37 GPa compared to 0.43–0.72 GPa), and higher HV (11.8–15.0 HV compared to 3.3–3.5 HV) than both of the methacrylate-based resins (KEY and CLE) after the production process. Although the TS, ME, and HV of the printed dimethacrylate resins (FRE, LUX, and VPR) decreased significantly under humid conditions with possibly elevated temperatures (thermocycling as well as 37 °C), these mechanical properties were significantly higher than both methacrylate-based resins (KEY and CLE). Therefore, printed dimethacrylate resins should be used rather than methacrylate-based resins for high expected masticatory forces, low wall thicknesses, or very long wearing times (≥6 months).

## 1. Introduction

Pain in the masticatory muscles and the temporomandibular joints, sometimes in combination with bruxism, are symptoms of temporomandibular joint dysfunction, which primarily affects young people and women [1,2]. Although clinical studies vary with regard to the treatment of pain, a placebo effect, and damage to the tooth structure [2,3,4], occlusal splints are a modern and recommended therapy [2,5]. In addition, occlusal splints are used for bite elevation in the course of a prosthetic restoration [6].

While a three-month observation period is standard, suggested wear durations vary considerably depending on the indication. For patients experiencing bruxism and painful masticatory muscles, nightly splint wear is recommended based on current research [7,8,9,10,11]. For patients who require a microdistraction splint or a splint for preprosthetic bite elevation, the daily wear time should be up to 24 h [8]. The materials used for this purpose must be able to withstand the oral cavity’s constant or intermittent stresses as well as the associated chemical and physical effects during the indication-specific wearing period. Despite their delicate design, they must be resistant to elastic deformation, have sufficient strength, and possess high wear resistance.

Subtractive CAD/CAM (computer-aided design/computer-aided manufacturing) technologies exhibit a higher degree of polymerization in comparison to conventional polymethacrylate splints due to the industrial manufacturing process of the pre-fabricated discs and the associated reduced polymerization shrinkage as well as improved biocompatibility and mechanics [6,12]. Three-dimensional (3D) printing is gaining importance as an additive manufacturing method due to its lower material consumption and simultaneously printable splints in the form of more resource-efficient and thus more cost-effective production. On the other hand, the supposed disadvantages can be the lower degree of polymerization and flaws in the microstructure, which can be avoided or minimized by optimized process technology, however [13].

Due to the relatively new process technology of 3D printing, there are currently only a few studies that deal with the mechanical properties of the new materials and the influence of aging processes on them. In addition, existing studies lack a uniform standardization of methodology, especially with respect to artificial aging [14,15]. Initial studies in the field of 3D plastics indicate that printed splints have comparable accuracy but exhibit higher material wear as well as less favorable material properties than their milled counterparts [16,17,18].

In addition to the manufacturing process, variations in monomer type frequently occur. Milled discs utilize MMA-based resins while printing processes employ DMA-based resins. Materials based on the polymerization of methyl methacrylate monomers differ substantially from those based on dimethacrylate monomers. The former only contain one polymerizable functional group. In contrast, the different dimethacrylate monomers contain two polymerizable functional groups on both ends of the monomer. Thus, they can crosslink upon polymerization [19]. The connecting part between the two methacrylate functional groups differs and can further tune the properties, e.g., the viscosity of the monomers [20].

The aim of this in vitro study was to compare different materials for 3D-printed or milled plastics for splint fabrication following an indication-specific wear time after artificial aging by dry, water, and thermal aging in terms of mechanical properties such as tensile strength, modulus of elasticity, and Vickers hardness. The null hypothesis of the study is:

**H_0_:**  *Material selection and aging processes have no influence on the mechanical properties of the occlusal splint materials in terms of tensile strength, elastic modulus, and Vickers hardness.*

The choice of simulation conditions made it possible to simulate specific indications in which the splint materials were used. According to the null hypothesis, the following points were to be evaluated for clinical use:

(Q1) *What influence does material selection have on the mechanical properties of occlusal splint materials?*
(Q2) *What is the relevance of dry or wet extraoral storage of the occlusal splint materials to the mechanical properties?*
(Q3) *Is there a difference in the mechanical properties of occlusal splint materials between nighttime and daytime use regarding cycling thermal storage?*

## 2. Materials and Methods

In the current study, four 3D-printed occlusal splint materials were investigated and compared with a milling material (Table 1) for tensile strength, modulus of elasticity, and Vickers hardness before and after different artificial aging processes.

### 2.1. Sample Preparation

A total of *n* = 75 specimens (ISO 527-2 type 1BA) [21] and *n* = 10 prisms (3 × 12 × 12 mm^3^) were virtually designed from each material (Netfabb 2020, Autodesk, Inc., San Francisco, CA, USA). The described specimens were produced from each resin (KEY, LUX, VPR, and FRE) using a 3D printer (Rapidshape P30, Straumann, Basel, Switzerland) according to the manufacturer’s instructions, cleaned in isopropanol, and post-exposed with 2 × 2000 light flashes under N2 protective atmosphere (SHERAflash-light plus, Shera Werkstoff-Technologie GmbH & Co. KG, Lemförde, Germany). Finally, the support structures were removed and the test specimens were polished using silicon carbide (SiC, P1200). For the production of the subtractively manufactured material (CLE), the corresponding digitally-designed test specimens were loaded as STL files into milling software (inLab v20, Dentsply Sirona Inc., Charlotte, NC, USA) and milled from industrially manu-factured blanks (inLab MC X5, Sirona, Germany). The post-processing step was equivalent to that of the printed test specimens.

### 2.2. Experimental Procedure

The prepared specimens were stored in water for seven days at 21 °C for pre-storage and randomly assigned into five groups. Group A (baseline) was analyzed and all other specimens were subjected to the respective storage protocol for groups B, C, D, and E (Table 2). Different loads were simulated during a six-month period of use, which were estimated according to the clinical wearing time (Table 2). Regarding group D, the approach chosen was 10,000 cycles (15 °C/35 °C/45 °C/35 °C), corresponding to the thermal load of one year [15]. The enlarged temperature change of 5 °C/55 °C, which is used in many in vitro experiments [22] as well as recommended by ISO 11405 [23], lowered the number of cycles, so we considered a load of 5 °C/55 °C for 2500 cycles as a representative value for the simulation of half a year of wearing time.

**Figure 1:**
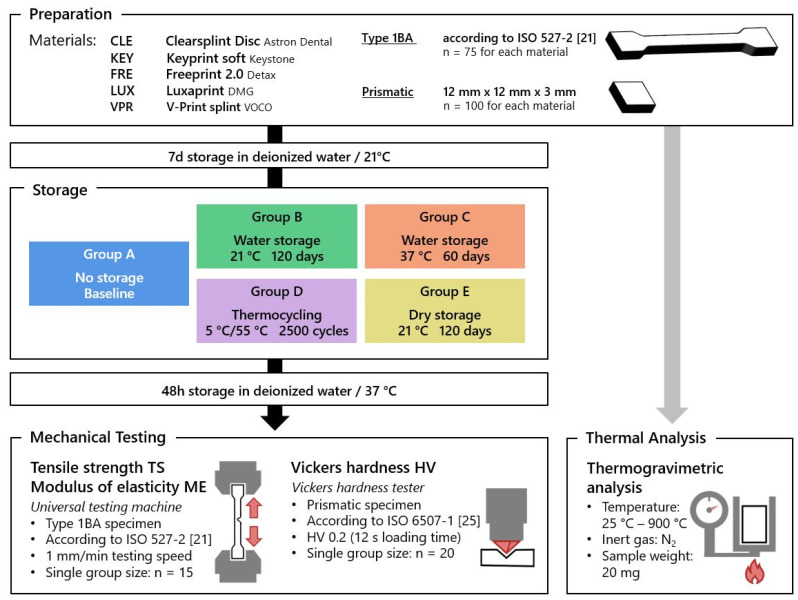
Study procedures with group A, water storage for 7 d at 21 °C (baseline); group B, water storage for 120 d at 21 °C; group C, water storage for 60 d at 37 °C; group D, thermocycling 2500 cycles at 5 °C/55 °C; group E, dry storage for 120 d at 21 °C. Tensile strength and modulus of elasticity based on tensile tests; Vickers hardness.

In order to ensure saturation directly before mechanical testing and a comparison between the storage types, 48 h water storage at 37 °C followed directly after the respective storage [24]. Finally, tensile strength, modulus of elasticity (Youngs modulus), and Vickers hardness were determined immediately (Figure 1).

### 2.3. Mechanical Properties

#### 2.3.1. Tensile Strength TS and Modulus of Elasticity ME

For each material and aging protocol, tensile strength and elastic modulus were tested on 15 specimens of type 1BA according to ISO 527-2 [21] using a universal testing machine (ZWICKRoell Retroline Z010, ZwickRoell GmbH & Co. KG, Ulm, Germany). For the determination of the modulus of elasticity, the specimens were loaded at a speed of 1 mm/min and the linear elastic deformation component was recorded with an external displacement transducer (ClipOn with a resolution of 1/1000 mm). The modulus of elasticity E_t_ was defined by the slope of the stress–strain curve σ(ε) in the strain range between ε_1_ = 0.05% and ε_2_ = 0.25%. After disassembly of the displacement transducer, the specimens were further loaded to failure at a rate of 1 mm/min up to the maximum failure load. The tensile strength σ_m_ was calculated from the maximum tensile stress and the cross-sectional area.

#### 2.3.2. Vickers Hardness HV

Vickers hardness (HV) was determined on plates (12 × 12 × 3 mm^3^) using a microindentation tester (MHT-4 Anton Paar, Graz, Austria) according to ISO 6507-1 [25] with a load of 0.2 kilopounds (HV0.2) and a loading time of 12 s [25]. For this purpose, the length of the diagonals of the created indentations (20 per group) was measured with a digital microscope (VK-X1000, Keyence, Osaka, Japan) and the Vickers hardness was calculated as follows:

HV = 0.1891 × F × d^−2^

F = testing force (N); d = diagonal length of indentation (mm).

### 2.4. Statistics

Calculations and graphical representations were performed using SPSS 29.0 (SPSS Corporation, Chicago, IL, USA). Since there were no normal distributions throughout all groups after the Shapiro–Wilk test, the data for tensile strength, modulus of elasticity, and Vickers hardness were first subjected to a directed rank transformation [26]. A two-way ANOVA was performed with the factors of material (PMMA and DMA) and storage (groups A, B, C, D, and E). The differences between the respective materials and groups of accelerated aging in terms of Vickers hardness, tensile strength, and modulus of elasticity were analyzed using Bonferroni post hoc analysis. The significance level was set at α = 0.05.

### 2.5. Thermogravimetric Analysis

Thermogravimetric analysis was performed on the basis of the mean sample mass of 19.4 ± 2.7 mg by means of a TGA/DSC1 STARe System (Mettler Toledo, Columbus, OH, USA) with corresponding software (STARe Software v14.0) using open corundum crucibles. The temperature interval ranged between room temperature to 900 °C with a heating rate of 10 K/min under nitrogen as inert gas with a gas flow of approx. 40 mL/min.

## 3. Results

The results of the two-way ANOVA are shown in Table 3. The material factor had a much greater influence than the bearing factor for the three mechanical properties. The subsequent sections delve into the distinct mechanical characteristics of the materials contingent on the storage prerequisites.

### 3.1. Tensile Strength (TS)

The statistical analysis shows that the tensile strength significantly relies on both the storage environment and materials used. However, the F-value indicated that the material was the decisive factor (see Table 3). Furthermore, prior to loading, the printed dimethacrylate resins (FRE, LUX, and VPR) were found to have a TS of 40–50 MPa compared to the milled (CLE) and printed (KEY) methacrylate resins with 12.3–13.3 MPa (Appendix A; Table A1).

**Figure 2:**
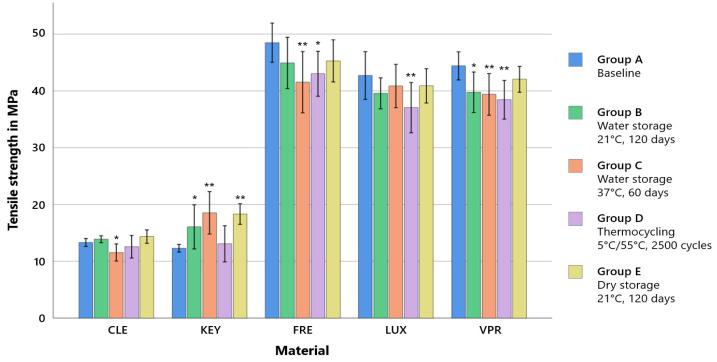
Mean tensile strength values (±SD) according to the storage conditions. ** strongly significant (*p* < 0.001) and * significant (*p* < 0.05) compared to the baseline of the same material.

The subtractively fabricated occlusal splint material (CLE) showed no significant changes in all other storage protocols (groups B, D, and E) except for a reduction in tensile strength after 60 days of water storage at 37 °C (group C). Contrary to this, a significant increase in tensile strength could be demonstrated with the printed product KEY both after dry (group E) and water storage (groups B and C) in contrast to thermocycling (group D). A significant decrease in tensile strength due to thermocycling (group D) was demonstrated for all printed dimethacrylate resins, FRE (48.5 MPa–43.0 MPa, *p* = 0.007), LUX (43.7 MPa–37.0 MPa, *p* < 0.001), and VPR (44.4 MPa–38.4, MPa *p* = 0.001). For VPR, a decrease in TS was observed for all longer water storage periods (group B: 39.8 MPa, *p* = 0.001; group C: 39.4 MPa, *p* < 0.001) (Figure 2; Appendix A, Table A1).

### 3.2. Modulus of Elasticity (ME)

With regard to the modulus of elasticity, only the material was found to be a significant factor (Table 3). The printed dimethacrylate resins (FRE, LUX, and VPR) exhibited a remarkably higher modulus of elasticity of 2.0 to 2.3 GPa compared to CLE with 0.4 to 0.5 GPa (*p* < 0.001) or KEY with 0.5 to 0.7 GPa.

**Figure 3:**
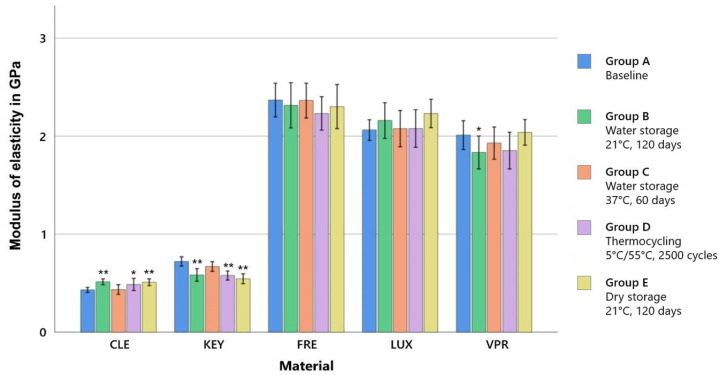
Mean values for the modulus of elasticity (± SD) according to the storage conditions, ** strongly significant (*p* < 0.001) and * significant (*p* < 0.05) compared to the baseline of the same material.

For the milled material CLE (baseline: 0.43 GPa), a significant increase in ME was observed relative to the baseline (0.43 GPa) except for group C, both upon dry storage (group E: 0.51 GPa, *p* < 0.001) and upon water storage for 120 d at 21 °C (group B: 0.51 GPa, *p* < 0.001), as well as by thermocycling (group D: 0.49 GPa, *p* = 0.005). In KEY, changes in ME (baseline: 0.7 GPa) occurred, after which, opposite to CLE, a decrease was observed under the same storage conditions (group E: 0.54 GPa, *p* < 0.001; group B: 0.58 GPa, *p* < 0.001; group D: 0.58 GPa, *p* < 0.001). The modulus of elasticity of the printed dimethacrylate resins (FRE, LUX, and VPR) showed no significant changes regarding the different storage protocols except for the storage of VPR in water (21 °C/120 days, *p* = 0.036) (Figure 3; Appendix A, Table A2).

### 3.3. Vickers Hardness (HV)

The ANOVA showed significant influence of the material and storage conditions, according to which the former is to be considered as the relevant factor due to a higher F-value. Already after pre-storage (baseline), the printed dimethacrylate resins (FRE, LUX, and VPR) showed higher Vickers hardness of FRE (11.8 HV), LUX (15.0 HV), and VPR (13.7 HV) compared to the methacrylate resins CLE (3.3 HV) and KEY (3.5 HV).

**Figure 4:**
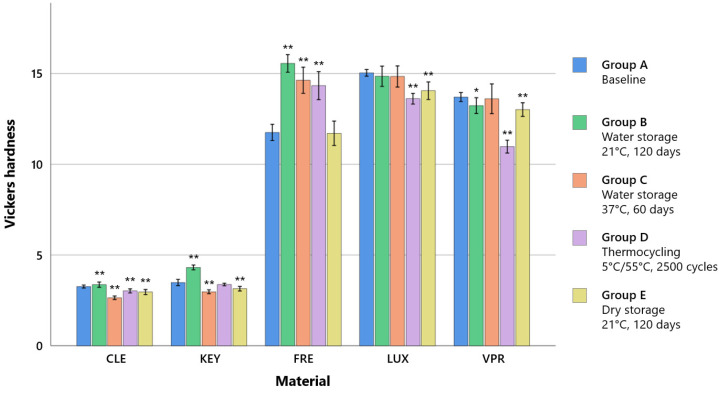
Mean Vickers hardness values (± D) according to the storage conditions. * significant (*p* < 0.05) compared to the baseline of same the material, ** strongly significant (*p* < 0.001) compared to the baseline of the same material.

After thermocycling (group D), a significant decrease in HV was observed for all occlusal splint materials (CLE: 3.0 HV, *p* < 0.001; FRE: 14.3 HV, *p* < 0.001; LUX: 13.6 HV, *p* < 0.001; VPR: 11.0 HV, *p* < 0.001), except for KEY (no change), and there was a significant increase in FRE (14.3 HV, *p* < 0.001). With the exception of FRE, there was a significant decrease in Vickers hardness for all materials when stored dry at 21 °C after 120 days (group E) (CLE: 3.0 HV, *p* < 0.001; FRE: 11.7 HV, *p* = 1; LUX: 14.0 HV, *p* < 0.001; VPR: 13.0 HV, *p* < 0.001) (Figure 4; Appendix A, Table A3).

## 4. Discussion

This study investigated the aging behavior of 3D-printed occlusal materials (KEY, FRE, LUX, and VPR) and a milled material (CLE) in terms of tensile strength (TS), modulus of elasticity (ME), and Vickers hardness (HV).

First and foremost, it could be observed that regardless of the aging scenario, the dimethacrylate-based resins (FRE, LUX, and VPR) exhibited remarkably higher values compared to the printed methacrylate-based material (KEY) and the milled reference material (CLE) for all investigated properties (TS, ME, and HV). This was supported by the results of the ANOVA, which defined the material as a decisive factor for the mechanical properties of the investigated occlusal splint materials.

In addition, different aging scenarios (Table 2) were simulated as they occur in real-life applications over a six-month period, and the different effects on the mechanical properties of the materials were observed. The results showed a significant reduction in the values for TS and HV for the printable dimethacrylate-based resins (FRE, LUX, and VPR) in thermal alternating storage (group D), which could be regarded as representative of the daily wearing time over half a year. Simulated overnight wear time (group C) resulted in a significant decrease in TS for CLE, FRE, and VPR and a significant decrease in Vickers hardness for CLE and KEY. Regarding ex situ storage, dry storage (group E) led to a significant decrease in hardness values in all material groups except for FRE, while wet storage (group B) led to a significant increase in hardness values in some material groups (CLE, KEY, and FRE).

The null hypothesis (H_0_) could be partially rejected.

In dental materials science, bending strengths (uniaxial and biaxial) are primarily compared, and minimum requirements are defined regarding this, e.g., for denture resins according to ISO 20795-1 with 65 MPa [27]. Flexural strength usually correlates with tensile strength and is considered the easier parameter to determine. Centric tensile tests with external deformation measurements are considered the more sensitive method because there are no transverse or compressive stresses in the specimen, only tensile stress. Therefore, in order to better detect the effects of aging, centric tensile tests were performed according to ISO 527-2 [21] rather than bending tests.

The higher the strength, the lower the risk of failure (fracture) due to masticatory loads while maintaining the same thickness. Alternatively, it is possible to produce thinner restorations (splints or removable protheses) with higher strengths. At the same time, a high modulus of elasticity is synonymous with low deformation behavior or rigid behavior under load. Bruxism patients in particular benefit from a rigid splint with a high strength [28,29,30], since their chewing loads can reach very high masticatory forces of up to 785 N [31].

Hardness is directly related to the modulus of elasticity of the material [32]. Hardness describes the irreversible deformability at the surface after constant loading and often correlates with the density of a material and its abrasion and/or scratch resistance [24].

(Q1) *What influence does material selection have on the mechanical properties of occlusal splint materials?*

**Figure 5:**
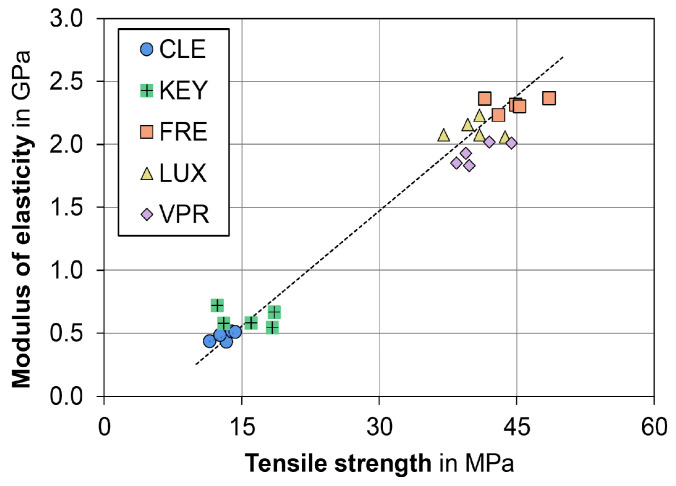
High (R^2^ = 0.98) correlation between tensile strength and modulus of elasticity for the two material groups: printed dimethacrylate resins (FRE, LUX, and VPR) and methacrylate resins (KEY and CLE). Each symbol shows a single storage condition.

**Figure A1:**
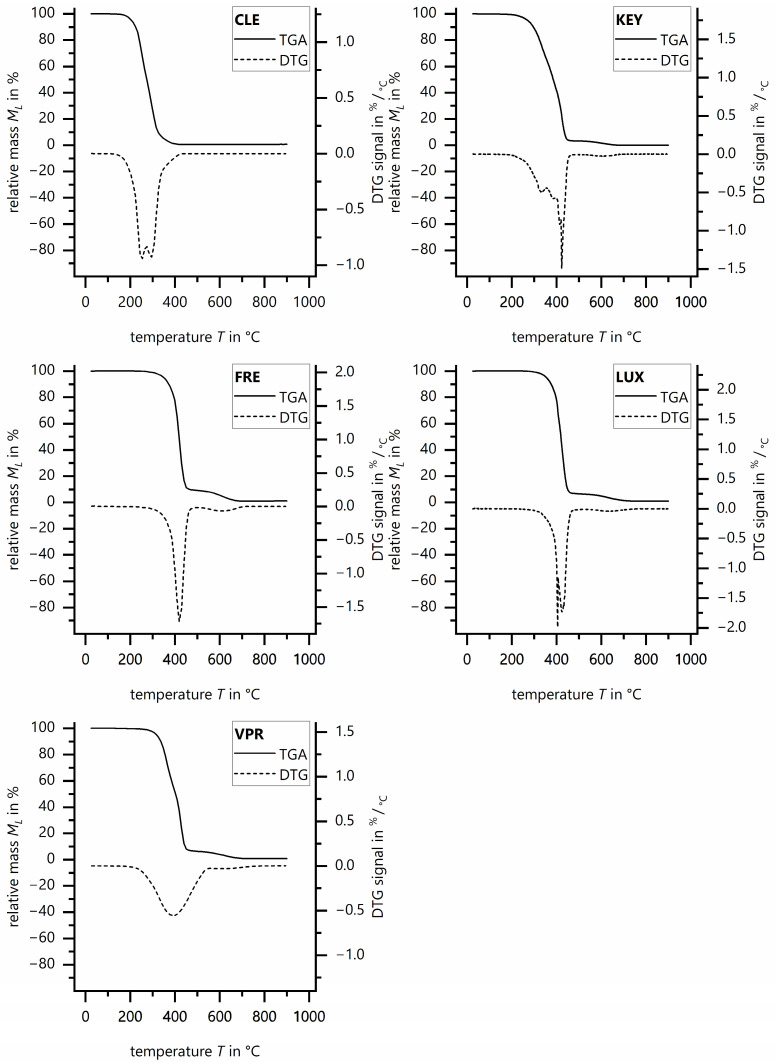
Thermogravimetric analysis and first derivative (DTG) between RT and 900 °C of Clearsplint (CLE), Keyprint (KEY), Luxaprint (LUX), V-Print splint (VPR), and Freeprint (FRE).

Material selection was a significant factor, that played a decisive role with regard to all tested mechanical properties. Storage conditions were also found to be a significant factor for tensile strength and Vickers hardness but played a minor role compared to material selection. The printed dimethacrylate resins (FRE, LUX, and VPR) showed higher tensile strength and modulus of elasticity (Young’s modulus) values by a factor of four due to a higher crosslinking (thermoset) than the two methacrylate-based resins (KEY and CLE, thermoplastic materials) (Figure 2 and Figure 3). The stress–strain analyses also showed that the methacrylate-based resins (KEY and CLE) had a ductile behavior and that the printed dimethacrylate resins had a more brittle behavior in comparison (Figure 5). The almost complete loss of mass at temperatures below 900 °C measured via thermogravimetric analysis was a clear indication that all investigated materials are free of inorganic fillers (see Appendix B, Figure A1). The mechanical effects were therefore determined by the polymer matrix. Important influencing factors were, for example, the type of monomers used, their interactions during copolymerization, their hydrophilicity, elasticity, and the strength and the degree of cross-linking, as well as the degree of polymerization [33,34]. Our investigations show the strong impact of the number of methacrylic functions per monomer, with the dimethacrylate-based materials (FRE, LUX, and VPR) investigated in our study having four-fold higher strength (Figure 5) and higher modulus of elasticity values (Figure 3) as well as higher Vickers hardness values (Figure 4) than the two methacrylate-based resins (CLE and KEY).

From a clinical point of view, the requirements for the material are stability, abrasion resistance (especially in the case of bruxism), a very good fit (neither a tight nor too loose fit), and adaptability. At the same time, the material should not be too soft so as not to increase chewing activity and thus muscle strain [35,36,37]. Stability can be described by the strength, abrasion resistance can be described by the hardness, and the deformation behavior can be described by the modulus of elasticity. The two common methacrylate resins (KEY and CLE) are considered to be rigid materials, so the printed dimethacrylate resins (FRE, LUX, and VPR) can also be categorized in this category due to their high modulus of elasticity (Figure 3). Due to their higher strength (Figure 2) and hardness (Figure 4), the printed dimethacrylate resins can be considered more stable. The higher modulus of elasticity means that the fit in particular is more important in the manufacturing process.

The tensile strength tends to change more than the modulus of elasticity due to the storage conditions. The production of the millable material (CLE) for industry use should have low levels of residual monomers (equivalent to a high polymerization) and smaller pore sizes compared to printed materials. Moreover, industrially manufactured materials typically exclude inhomogeneously polymerized layers, as they can cause modified fracture behavior [38]. It can be deduced that, given identical monomers, the milled materials are more advantageous than the printed materials regarding potential leaching and degradation effects [39]. Furthermore, it cannot be ruled out that the printed methacrylate resins (KEY) underwent post-polymerization, as the tensile strength increased after being stored for longer periods (>60 days) in groups B, C, and E. The relatively uniform values of strength (refer to Appendix A, Table A1) observed in the milled material (CLE) as compared to the printable resins (KEY, LUX, VPR, and FRE) support this hypothesis. However, differences could still have arisen due to the presence of various monomers. For instance, Szczesio-Wlodarczyk et al. (2021) demonstrated that copolymers made of distinct dimethacrylate monomers differ in their water absorption and solubility [34]. Consequently, adsorption leads to the hydrolytic degradation of the resin matrix, causing mechanical property deterioration [40].

The tensile strengths and hardnesses of the printed dimethacrylate resins (LUX, VPR, and FRE) decreased significantly (*p* < 0.05) following thermocycling (group D) and after water storage at 37 °C (group C). Only LUX did not show a significant decrease in strength under the storage conditions and without the influence of thermocycling (groups B, C, and E). The reason for this could be a high degree of polymerization and a low solubility. The low water sorption in the polymer matrix could be associated with a lower susceptibility to hydrolytic degradation. However, the high content of bisphenol A diglycidyl methacrylate (Bis-EMA)—a hydrophobic monomer with low water absorption and dissolution—is not sufficient as an absolute explanation, since this monomer is also present in VPR and FRE [34]. Lower proportions or the absence of hydrophilic monomers (such as triethylene glycol dimethacrylate = TEGDMA in VPR) may also play a role here.

For the milled thermoplastic material (CLE), as expected, the storage type had no significant influence on the change in tensile strength. On the other hand, the compressible thermoplastic (KEY) exposed to elevated temperature (37 °C in water, group C) or dry conditions (group E) showed a significant increase in tensile strength but a lower modulus of elasticity. The increase in strength at high temperatures could be explained by post-polymerization. Urban et al. (2009) reported increased degrees of polymerization and improved mechanical properties through post-polymerization (water bath, 55 °C, 10 min) for some of the methacrylate-based hard chairside reline resins they investigated [41].

Perea-Lowery et al. (2021) were able to improve the degree of polymerization and the mechanical properties (flexural strength, fracture toughness, and surface hardness) of a dimethacrylate-based resin for the 3D printing of occlusal splints by using light in combination with heat (60 °C) [42]. Water saturation occurs even in industrially produced thermosets with very few flaws [43] even after 90 to 180 days [44] and often results in the deterioration of mechanical properties [17,45,46]. However, increased porosity or increased solubility to mobilize the monomers, as discussed by Berli et al. (2020) [17], is not mandatory for the deterioration of the mechanical properties. The decreasing tensile strength of all dimethacrylate-based resins compared to the reference storage and the partly increasing hardness during humid storage suggested that water saturation has a much greater influence on the mechanical properties than the post-polymerization process mentioned in the literature.

(Q2) *What is the relevance of dry or wet extraoral storage of the occlusal splint materials to the mechanical properties?*

Extraoral storage (group B + group E) showed occasional minor significances depending on the storage medium (wet or dry). While extraoral dry storage had no significant influence on the TS and ME of the printed dimethacrylate-based resins (FRE, LUX, and VPR), a decrease in strength and elastic modulus was observed for VPR for the simu-lation of extraoral storage in water. For the milled methacrylate resin, extraoral storage (dry: group E; wet: group B) did not influence the strength, while an increase in the elastic modulus was observed (Figure 2 and Figure 3).

With regard to hardness, the printed dimethacrylate-based resins showed lower values due to dry storage (group E) than after water storage (group B). The increasing hardness could be related to the water absorption and associated swelling behavior (see discussion Q1). The two thermoplastic materials (KEY and CLE), on the other hand, exhibited minor changes with respect to the two storage conditions (see Appendix A, Table A3). Accordingly, no general recommendation could be made regarding the extraoral storage conditions, independent of the material and the mechanical properties.

(Q3) *Is there a difference in the mechanical properties of occlusal splint materials between nighttime and daytime use regarding cycling thermal storage?*

Most indications for wearing occlusal splints are described as sufficient with nightly wearing at 37 °C body temperature (cf. group C), when no temperature fluctuations due to food intake are to be expected [7,9,11]. In addition, thermocycling (group D) was another indication included in this study, which involved 16 h of wear over half a year [8,15].

While both aging simulations significantly lowered the strength/modulus of elasticity of the printed dimethacrylate resins (FRE, LUX, and VPR), thermocycling and wet storage at 37 °C of the methacrylate-based resins (KEY and CLE) did not have a negative effect or had minimal impact on the mechanics. Due to the lower strength level of methacrylate-based resins (tensile strength 2.6–3.5 MPa) compared to printed dimethacrylate resins (11.0–8.8 MPa), before but also after aging, greater thicknesses for occlusal splint materials have to be chosen when using methacrylate-based resins. This means that printed dimethacrylate resins can endure a longer service life and greater mechanical loads with an equivalent layer thickness.

### Study Limitations and Future Prospects

The study design was chosen to analyze fundamental differences in material behavior before and after aging. Therefore, the study is limited to the in vitro characteristics, which do not take into account the various clinical situations with different loading conditions and different design geometries. The results and discussion presented are limited to the subject of the study, so general statements can only be made with further investigations. Future studies should also simulate the cyclic mechanical stresses that occur in real life with craniomandibular dysfunction.

## 5. Conclusions

Based on the tensile strengths, modulus of elasticity and Vickers hardnesses measured in this study, the following conclusions were drawn:

(1) Printed dimethacrylate resins (FRE, LUX, and VPR)—capable of forming a crosslinked matrix—show significantly higher tensile strengths (43.7–48.5 MPa compared to 12.3–13.3 MPa), modulus of elasticity (2.0–2.4 GPa compared to 0.4–0.7 GPa), and hardness (11.8–15.0 HV compared to 3.3–3.5 HV) than printed or milled methacrylate resins (CLE and KEY). No significant difference in strength and hardness was found between printed and milled methacrylate resins.
(2) The mechanical performance of printed dimethacrylate resins (FRE, LUX, and VPR) deteriorated significantly under humid conditions with high temperatures (thermocycling as well as 37 °C). However, despite the performance loss, the mechanical properties are still significantly superior to those of the methacrylate-based resins (CLE and KEY).
(3) None of the specimens failed due to thermal aging in a humid environment. Accordingly, all materials can be used clinically for at least six months without concern. In the case of high expected chewing forces or low material thicknesses, printed dimethacrylates should be used rather than methacrylate-based resins due to their better mechanical properties.

## Figures and Tables

**Table 1:** Overview of materials with fabrication (F: P—printed at 385 nm wavelength, 90°, and M—milled with 50,000 U/min) and composition (manufacturer’s information).

F	CODE	Product	Manufacturer	LOT	Composition	
M	CLE	CLEAR splint Disc	Astron Dental Corporation, Lake Zurich, IL, USA	E71342-12/86520 E71350-5/86523	Poly(ethyl methacrylate), 2-ethoxyethyl methacrylate, and dibenzoyl peroxide	MA
P	KEY	KeyPrint KeySplint soft	Keystone Industries, Gibbstown, NJ, USA	JK7893	2-phenoxyethyl methacrylate, isobornyl methacrylate, 2-hydroxyethyl methacrylate, and TPO (=diphenyl(2,4,6-trimethylbenzoyl)phosphine oxide)	MA
P	FRE	FREEPRINT^®^ splint 2.0	Detax GmbH & Co KG, Ettlingen, Germany	220807	Isopropylidenediphenol peg-2 dimethacrylat (90–<95%), 2-propenoic acid, (5-ethyl-1,3-dioxan-5-yl)methyl ester (1–<5%), and diphenyl (2,4,6-trimethylbenzoyl)phosphine oxide (1–<5%)	DMA
P	LUX	Luxaprint Ortho Plus	DMG GmbH, Hamburg, Germany	201588	EBPADMA (=ethoxylated Bisphenol A dimethacrylate) (>90%) and diphenyl(2,4,6-trimethylbenzoyl)phosphine oxide (1–2%)	DMA
P	VPR	V-Print splint	Voco GmbH, Cuxhaven, Germany	1942592	Polyester dimethacrylate, BIS-EMA (=ethoxylated Bisphenol A dimethacrylate), triethylenglycol dimethacrylate, hydroxypropyl methacrylate, diphenyl(2,4,6-trimethylbenzoyl)phosphine oxid, and BHT (=butylated hydroxytoluene)	DMA

**Table 2:** Reality simulation and storage protocols.

Group	Storage Protocol	Reality Simulation
A	Baseline (0 d)	Reference
B	Water storage for 120 d at 21 °C	Half-year (16 h/d) extraoral wet storage
C	Water storage for 60 d at 37 °C	Half-year (8 h/d) at night in the mouth
D	Thermocycling of 2500 cycles at 5 °C/55 °C	Half-year (16 h/d) daytime usage
E	Dry storage for 120 d at 21 °C	Half-year (16 h/d) extraoral dry storage

**Table 3:** Results of the two-way ANOVA.

Factor	Tensile Strength		Modulus of Elasticity		Vickers Hardness	
	F-Value	*p*-Value	F-Value	*p*-Value	F-Value	*p*-Value
Storage	10.937	<0.001	2.386	0.051	41.916	<0.001
Material	942.641	<0.001	938.864	<0.001	1268.712	<0.001
Storage × Material	11.879	<0.001	2.042	0.088	17.346	<0.001

## Data Availability

All data presented in this study are available in the article and in the Appendix A and Appendix B.

## Appendix A

**Table A1:** Means and standard deviations of tensile strength according to the storage conditions; capital letters indicate a significant difference (*p* < 0.05) compared to the respective storage condition for each material.

	Tensile Strength/MPa				
Materials	A	B	C	D	E
	Baseline	120 d Water Storage 21 °C	60 d Water Storage 37 °C	Thermocycling	120 d Dry Storage 21 °C
Clearsplint (CLE)	13.3 ± 0.7 ^C^	13.9 ± 0.6 ^C^	11.5 ± 1.5 ^ABE^	12.6 ± 2.0 ^E^	14.3 ± 1.2 ^CD^
Keyprint (KEY)	12.3 ± 0.7 ^BCE^	16.0 ± 3.9 ^A^	18.5 ± 3.7 ^AD^	13.0 ± 3.2 ^CE^	18.3 ± 1.8 ^AD^
Freeprint 2.0 (FRE)	48.5 ± 3.4 ^CD^	44.9 ± 4.5	41.5 ± 5.4 ^A^	43.0 ± 4.0 ^A^	45.3 ± 3.7
Luxaprint (LUX)	43.7 ± 4.2 ^D^	39.6 ± 2.7	40.9 ± 3.8	37.0 ± 4.4 ^A^	40.9 ± 3.0
V-Print splint (VPR)	44.4 ± 2.5 ^BCD^	39.8 ± 3.6 ^A^	39.4 ± 3.7 ^A^	38.4 ± 3.4 ^AE^	42.0 ± 2.9 ^D^

**Table A2:** Means and standard deviations of elastic modulus according to the storage conditions; capital letters indicate a significant difference (*p* < 0.05) compared to the respective storage condition for each material.

	Modulus of Elasticity/GPa				
Materials	A	B	C	D	E
	Baseline	120 d Water Storage 21 °C	60 d Water Storage 37 °C	Thermocycling	120 d Dry Storage 21 °C
Clearsplint (CLE)	0.43 ± 0.03 ^BDE^	0.51 ± 0.03 ^AC^	0.44 ± 0.05 ^BDE^	0.49 ± 0.06 ^AC^	0.51 ± 0.04 ^AC^
Keyprint (KEY)	0.72 ± 0.05 ^BDE^	0.58 ± 0.06 ^AC^	0.67 ± 0.05 ^BDE^	0.5 ± 0.05 ^AC^	0.55 ± 0.05 ^AC^
Freeprint 2.0 (FRE)	2.37 ± 0.17	2.31 ± 0.23	2.36 ± 0.18	2.23 ± 0.17	2.30 ± 0.23
Luxaprint (LUX)	2.06 ± 0.11	2.16 ± 0.18	2.08 ± 0.19	2.08 ± 0.19	2.23 ± 0.15
V-Print splint (VPR)	2.01 ± 0.15 ^B^	1.83 ± 0.17 ^AE^	1.93 ± 0.16	1.85 ± 0.19 ^E^	2.02 ± 0.13 ^BD^

**Table A3:** Means and standard deviations of Vickers hardness according to the storage conditions; capital letters indicate a significant difference (*p* < 0.05) compared to the respective storage condition for each material.

	Vickers Hardness HV0.2				
Materials	A	B	C	D	E
	Baseline	120 d Water Storage 21 °C	60 d Water Storage 37 °C	Thermocycling	120 d Dry Storage 21 °C
Clearsplint (CLE)	3.3 ± 0.1 ^CDE^	3.4 ± 0.1 ^CDE^	2.6 ± 0.1 ^ABDE^	3.0 ± 0.1 ^ABC^	3.0 ± 0.1 ^ABC^
Keyprint (KEY)	3.5 ± 0.2 ^BCE^	4.3 ± 0.1 ^ACDE^	3.0 ± 0.1 ^ABDE^	3.4 ± 0.1 ^BCE^	3.1 ± 0.1 ^ABCD^
Freeprint 2.0 (FRE)	11.8 ± 0.5 ^BCD^	15.6 ± 0.5 ^ACDE^	14.6 ± 0.7 ^ABE^	14.3 ± 0.8 ^ABE^	11.7 ± 0.7 ^BCD^
Luxaprint (LUX)	15.0 ± 0.2 ^DE^	14.9 ± 0.6 ^DE^	14.8 ± 0.6 ^DE^	13.6 ± 0.3 ^ABCE^	14.1 ± 0.5 ^ABCD^
V-Print splint (VPR)	13.7 ± 0.3 ^BDE^	13.2 ± 0.4 ^AD^	13.6 ± 0.8 ^DE^	11.0 ± 0.4 ^ABCE^	13.0 ± 0.4 ^ACD^
